# Development and validation of a deep learning ultrasound radiomics model for predicting drug resistance in lymph node tuberculosis a multicenter study

**DOI:** 10.1097/JS9.0000000000002850

**Published:** 2025-07-02

**Authors:** Xu Zhang, Zhijian Dong, Hongming Li, Yijing Cheng, Wei Tang, Tu Ni, Ying Zhang, Qinqin Ai, Gaoyi Yang

**Affiliations:** a Department of Ultrasonography, Hangzhou Red Cross Hospital, Hangzhou, Zhejiang, China; b Department of Ultrasonography, Kunming Third People’s Hospital, Kunming, Ynunan, China; c Department of Ultrasound, Heilongjiang Infectious Disease Prevention and Treatment Hospital, Harbin, China; d Department of Hepatology, Hangzhou Xixi Hospital, Hangzhou, China; e Department of Ultrasound, Affiliated Hangzhou First People’s Hospital, School of Medicine, Westlake University, Hangzhou, China

## Abstract

**Background:**

To develop and validate an ensemble machine learning ultrasound radiomics model for predicting drug resistance in lymph node tuberculosis (LNTB).

**Materials and methods:**

This multicenter study retrospectively included 234 cervical LNTB patients from one center, randomly divided into training (70%) and internal validation (30%) cohorts. Radiomic features were extracted from ultrasound images, and an L1-based method was used for feature selection. A predictive model combining ensemble machine learning and AdaBoost algorithms was developed to predict drug resistance. Model performance was assessed using independent external test sets (Test A and Test B) from two other centers, with metrics including AUC, accuracy, precision, recall, F1 score, and decision curve analysis.

**Results:**

Of the 851 radiometric features extracted, 161 were selected for the model. The model achieved AUCs of 0.998 (95% CI: 0.996–0.999), 0.798 (95% CI: 0.692–0.904), 0.846 (95% CI: 0.700–0.992), and 0.831 (95% CI: 0.688–0.974) in training, internal validation, and external test sets A and B, respectively. The decision curve analysis showed a substantial net benefit across a threshold probability range of 0.38 to 0.57.

**Conclusion:**

The LNTB resistance prediction model developed demonstrated high diagnostic efficacy in both internal and external validation. Radiomics, through the application of ensemble machine learning algorithms, provides new insights into drug resistance mechanisms and offers potential strategies for more effective patient treatment.

**Keywords:**

drug resistance, lymph node tuberculosis, machine learning, radiomics, ultrasound

## Introduction

Lymph node tuberculosis (LNTB) is the most common extra-pulmonary tuberculosis, and lymph nodes in all regions of the body can be involved^[1,2]^. In recent years, drug resistance to Mycobacterium tuberculosis (MTB) has become increasingly serious, in particular the prevalence and spread of multidrug-resistant tuberculosis and extensively drug-resistant tuberculosis, which have a long treatment cycle, difficult control and low cure rate, posing a serious threat to global tuberculosis prevention and control^[3-5]^. According to the WHO Global Tuberculosis Report 2023, 3.3% of new TB cases and 17% of previously treated patients had MDR/rifampicin-resistant TB. An estimate of drug resistance is extremely important in the epidemiology and control of MTB^[6-8]^.

**KEY POINTS**

- Accurate prediction of LNTB resistance is crucial for treatment planning.
- The LNTB drug-resistance prediction model showed high diagnostic efficacy.
- Multicenter research can increase representativeness and generalization ability.

**HIGHLIGHTS**

- Combining ultrasound radiomics with ensemble machine learning algorithms can effectively predict drug resistance in lymph node tuberculosis (LNTB).
- This method exhibits high diagnostic efficiency and has potential for clinical application.
- Future research should focus on further validating the model’s generalizability and exploring additional modalities of ultrasound image data to enhance predictive accuracy.

Currently, detection of resistance to LNTB relies on culture and drug sensitivity testing of MTB in biopsy tissue. However, the lengthy culture period and low positive rate of MTB pose considerable challenges in clinical diagnosis and treatment. With its high sensitivity and specificity, the Xpert/RIF test can not only diagnose MTB but also detect drug resistance. While the Xpert/RIF test offers rapid diagnosis and detection of Rifampicin resistance, other molecular techniques still require further development. For instance, whole genome sequencing (WGS) and line probe assays (LPA) provide a thorough understanding of the genetic underpinnings of drug resistance, as WGS costs decline and increasingly rival traditional diagnostic methods. This advancement could enhance the clinical utility of WGS, making it a viable alternative to current standard tests, especially in the context of acute leukemia diagnosis^[9,10]^. Furthermore, lymph node biopsy represents an invasive diagnostic procedure, puncture will cause injury, and may lead to MTB spread and sinus formation^[11]^. Therefore, the question arises whether LNTB resistance can be detected through non-invasive tests, which is of utmost importance.

Radiomics is a process of extracting quantitative feature information in a high-throughput manner from acquired images, converting image images into high-dimensional data to achieve quantitative analysis of image information and capturing a large number of lesion heterogeneity information that cannot be observed by the naked eye^[12,13]^. It has demonstrated outstanding performance in tumor classification, diagnosis and prediction. However, there are few domestic and foreign reports on ultrasound radiomics in LNTB drug resistance detection^[14,15]^. This approach has been successfully applied in various infectious diseases, including tuberculosis, to provide insights into disease heterogeneity and predict treatment outcomes^[16,17]^.

The objective of this research is to analyze ultrasound images of LNTB employing the ultrasound radiomics approach, identify the distinctive characteristics of drug-resistant versus non-drug-resistant LNTB, and construct a predictive model leveraging these differential characteristics through an ensemble machine learning algorithm, thereby achieving a non-invasive diagnostic method for distinguishing between drug-resistant and non-drug-resistant LNTB. This study adheres to the Transparency In The Reporting of Artificial Intelligence (TITAN) guidelines^[18]^, ensuring transparency in the application of ensemble machine learning algorithms.

## Materials and methods

### Study cohorts

The study was a multicenter diagnostic study using independent data provided by three hospitals in China. Baseline characteristics of the participants encompassed age, sex, lymph node status, histology type, and final diagnosis, excluding race data. The study was approved by the Ethics Committee used by the Participating Center (2022 Review No.121). This study complies with China’s 2023 Ethical Review Measures for Human Life Sciences and Medical Research. For anonymized ultrasound images collected retrospectively, informed consent was waived. No additional follow-up or compensation was involved in this study. In the prospective dataset, participants clearly understood and agreed that their clinical information, such as lymph node images, age, gender, etc., would be used for the study publication and approved by the ethics committee. The report of this study was written following the guidelines outlined in the STARD statement^[19]^.

**Figure 1.**
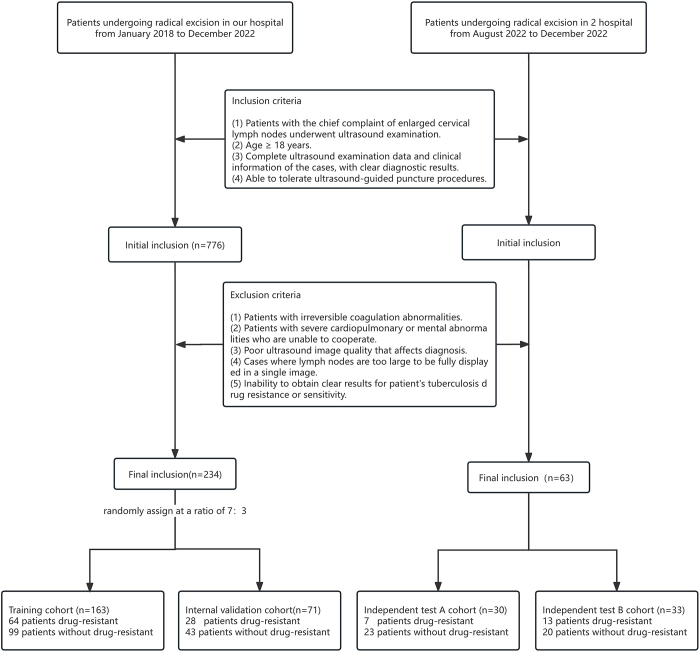
Flow chart of the study population selection strategy.

Retrospective clinical data were collected from Hangzhou Red Cross Hospital between January 2018 and December 2022. These LNTB ultrasound images were included in the study and randomly assigned to the training and validation sets in a 7:3 ratio. At the same time, the prospective dataset was derived from ultrasound images of lymph nodes at Kunming Third People’s Hospital (Test A) and Heilongjiang Provincial Infectious Disease Hospital (Test B) between August 2022 and December 2023, which were used as external test sets for the evaluation of the model. Figure 1 shows a flowchart of the studied population.

The inclusion criteria: (1) patients who underwent ultrasound examination for cervical lymph node enlargement; (2) age ≥8 years old; (3) complete ultrasound data and clinical information of cases, with clear diagnostic results.

Exclusion criteria: (1) patients with uncorrectable coagulation dysfunction; (2) patients with severe cardiopulmonary dysfunction or mental abnormality who are unable to cooperate; (3) poor ultrasound image quality, affecting diagnosis; (4) large lymph nodes that cannot be fully displayed in one image.

Diagnostic criteria: pathological diagnosis serves as the gold standard for diagnosis. Cases that could not be confirmed by histological analysis were comprehensively analyzed by two senior respiratory physicians based on imaging findings, microbiological evidence, and clinical symptoms.

### Ultrasound radiomics feature extraction and ultrasound image segmentation

Retrospective ultrasound images were normalized and gray values were converted to standardized intensity ranges. For prospectively collected data, multicenter studies were conducted using standardized protocols for ultrasound devices, including probe frequencies (such as uniform use of 7-12/5-9 MHz linear probes), gain adjustments, and image storage formats. Then, select gray-scale sonography biggest section of ultrasound images, lymph nodes and import the 3D slicer software (version Slicer – 4.11.20200930, https://download.slicer.org/). Pyradiomics^[20]^ is an open source Python package specifically designed to extract radiomics features from medical images. Detailed information on the extraction and processing of radiomics features is provided in Supplementary Digital Content, Material 1 (available at: http://links.lww.com/JS9/E491). ROIs were manually delineated by two independent sonographers (X.Z. and Y.Z., with >5 years of experience in lymph node ultrasound) using 3D Slicer software, operating blindly to clinical outcomes. For cases with inter-observer ROI overlaps <85%, a third senior sonographer (W.T.) resolved differences through consensus annotation.

### Radiomics feature screening through inter-observer consistency (ICC) evaluation

Inter-observer ICC values were calculated for all 851 features, and 39 features with ICC <0.75 were excluded from model construction. Inter-observer consistency evaluation of ultrasound image segmentation is shown in Supplementary Digital content, Material 2 (available at: http://links.lww.com/JS9/E492).

### Radiomics feature screening

The high-dimensional data extracted through radiomics cannot be directly utilized in its entirety for model development. Instead, features exhibiting the most significant variations must be rigorously filtered to construct robust predictive models. A linear model-based L1 regularization approach is employed for streamlined feature screening. By constructing a robust linear model on training data, this method derives a sparse coefficient matrix through strategic regularization. The stringency of feature filtration is precisely calibrated via parameter C – the lower the value of C, the more stringent the feature selection becomes. Optimized parameter tuning at 0.1C effectively preserves features with significant coefficient magnitudes. This intelligent screening mechanism not only extracts the most predictive features, but also bolsters the model’s interpretability while enhancing its generalization capabilities.

### Construction of ensemble machine learning prediction model

This study employs an ensemble machine learning framework, where radiomics feature extraction serves as a data-driven quantitative analysis process, and AdaBoost acts as an ensemble classification algorithm for model construction. Feature extraction relies on 3D Slicer software and PyRadiomics library, while classification is achieved through AdaBoost’s iterative enhancement of weak classifiers, synergistically completing the full-process modeling from images to drug resistance prediction. Prior to choosing AdaBoost model, several other machine learning models were evaluated using performance metrics such as accuracy, precision, recall, and F1 score.

The AdaBoost model was chosen due to its superior performance in classifying drug-resistant and drug-sensitive LNTB cases. A diagnostic model was established by using Rad-score (Radiomics Score) and AdaBoost algorithm of ensemble machine learning to predict drug resistance in neck LNTB patients. The AdaBoost algorithm is an ensemble learning method that builds a strong classifier by combining several weak classifiers. Specifically, we use the entire training set to train the weak learning machine and redistribute the weight of the sample in each iteration. According to the error of the previous weak classifier, we use a weighted way to build a more powerful classifier. Each new weak classifier is trained on the training set and generates new sample weights and weak classifier weights. This iterative process continues until a predetermined error rate is reached or the specified maximum number of iterations is reached. Through this iterative process, AdaBoost algorithm can gradually improve the performance of the classifier model, thus improving the classification accuracy. In this study, we chose Rad-score as a feature and combined it with AdaBoost algorithm to construct a diagnostic model specifically designed to predict and classify drug resistance in patients with neck LNTB.

### Validation and evaluation of predictive models

An internal validation queue and two external test queues were used to evaluate the diagnostic efficiency of the predictive model constructed by radiomics. An internal validation queue is a validation performed on a training dataset to evaluate the performance of a model on a relatively small sample set. The external test queue consists of independent sample sets that are used to evaluate the model’s generalization capability.

### Statistical method

R software (Version 3.5.3, http://www.rproject.org) was used for data analysis and mapping. Count data were expressed as example (%), and χ2 test or Fisher exact probability method were used for comparison between groups. Measurement data followed normal distribution and were expressed as mean ± standard deviation. Two independent T-test samples were used for comparison between groups. The Mann-Whitney U test was used to compare non-normal distribution measurement data. To address overfitting, nested cross-validation (5 inner folds, 3 outer folds) was applied during model training, and L2 regularization with a penalty parameter of λ = 0.01 was incorporated into the AdaBoost algorithm to limit model complexity. The diagnostic efficiency of the predictive model is evaluated using the Area Under Curve (AUC) of the receiver operating characteristic curve (ROC), and the ACC of the predictive model is calculated. Precision, Recall, F1 score. To further evaluate the clinical application value of the predictive model, the net benefit of different threshold probabilities was quantified using Decision Curve Analysis (DCA). The test level was *P* <0.05.

### Role of the funding source

The funders of the study had no role in the study design, data collection, data analysis, data interpretation, or writing of the report.

## Results

### Clinical characteristics of patients in drug-resistant and sensitive groups

A total of 234 patients with lymph node tuberculosis were included in the study. The model training data set and internal validation set were independent of each other, including 92 cervical LNTB patients in the drug-resistant group, including 34 males and 58 females, aged 19-79 years (33.8 ± 15.1 years). Of 142 patients with neck LNTB in the drug-sensitive group, 61 were males and 81 were females, ranging in age from 17 to 87 years (41.2 ± 18.3 years). There was no significant difference in age and gender between the two groups (*P* > 0.05), as shown in Table 1.

**Table 1:** Patient’s characteristics at baseline

	Overall group (n = 234)			Test A (n = 30)			Test B (n = 33)		
Variable	Drug sensitive	Drug resistance		Drug sensitive	Drug resistance		Drug sensitive	Drug resistance	
	(n = 142)	(n = 92)	*P* value	(n = 23)	(n = 7)	*P* value	(n = 20)	(n = 13)	*P* value
**Age (y)**	33.8 ± 15.1	41.2 ± 18.3	0.266	39.8 ± 9.4	40.5 ± 13.4	0.439	43.3 ± 11.1	39.9 ± 12.3	0.577
**Sex**									
Male	61(43.0%)	34(37.0 %)	0.361	9(39.1%)	5(71.4 %)	0.204*	7(35.0 %)	5(38.5%)	0.999*
Female	81(57.0%)	58(63.0%)		14(60.9%)	2(28.6%)		13(65.0 %)	8(61.5%)	

^*^Fisher exact probability method.

All samples were randomly assigned in a 7:3 ratio to the training and validation cohorts. The training cohort (163 patients) included 99 drug-sensitive patients and 64 drug-resistant patients. The validation cohort (71 patients) included 43 drug-sensitive patients and 28 drug-resistant patients.

A total of 30 patients were enrolled in cohort A, including 7 patients with resistance and 23 patients with sensitivity. There were 14 males and 16 females, ranging in age from 22 to 72 years (35.9 ± 13.1 years).

A total of 33 patients were enrolled in cohort B, including 13 patients with resistance and 20 patients with sensitivity There were 12 male and 21 female LNTB patients in the group, ranging in age from 20 to 73 years (36.2 ± 19.3 years).

### Consistency test of radiomics features

Image radiomics features were extracted from 234 gray scale images, and a total of 851 features were extracted. In the consistency assessment, the ICC was employed to evaluate the repeatability of radiomics parameter measurements during observer ROI mapping. To ensure robust consistency in the repeatability outcomes, 39 features demonstrating ICC values below the 0.75 threshold were ultimately excluded from model construction, as detailed in Supplementary Digital Content, Material 2 (available at: http://links.lww.com/JS9/E492).

Feature screening was conducted through in two sequential phases. Initially, variance filtering was implemented, excluding features demonstrating variance below 20% of the training set mean – a data-driven threshold – which eliminated 173 low-variability features. Subsequently, Pearson’s correlation analysis was applied to the remaining features. Feature pairs exhibiting |r| > 0.9 were deemed highly correlated; within each such pair, the feature with reduced variance was systematically pruned, culminating in 517 exclusions. This dual phase approach strategically balances feature variability and collinearity, thus preserving statistically discriminative features. The model was subsequently trained using the refined set of 161 features. For insights into the predictive capacity of highly correlated features, refer to Supplementary Digital Content, Material 3 (available at: http://links.lww.com/JS9/E493).

### Construction of radiomics model

**Figure 2.**
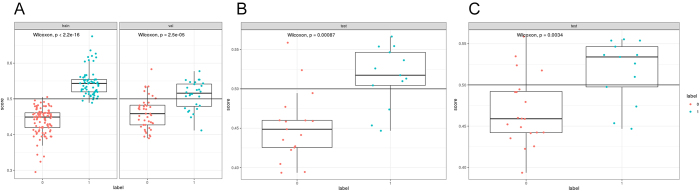
Box plot shows stratification of radiomics signature based on LNTB drug resistance.

In this study, the AdaBoost model with the best classification effect was selected to classify and diagnose LNTB resistance in neck, and the categories of drug resistance and sensitivity of samples were obtained (Fig. 2).

(A) Training set and internal validation set, (B) external validation set (Test A), and (c) external validation set (Test B), respectively.

The AdaBoost classification algorithm was used to construct the model through transfer learning. Model superparameter setting: {“n_estimators”: 150, “learning_rate”: 0.1, “algorithm”: “SAMME.R”}, fit_pred_score = 0.681, for random state = 1. The diagnostic value distribution map of the drug-resistant LNTB imaging model is presented in the form of charts, which more intuitively shows whether the imaging model diagnoses the neck LNTB patients with drug resistance, as well as the contribution and change of each component.

**Figure 3.**
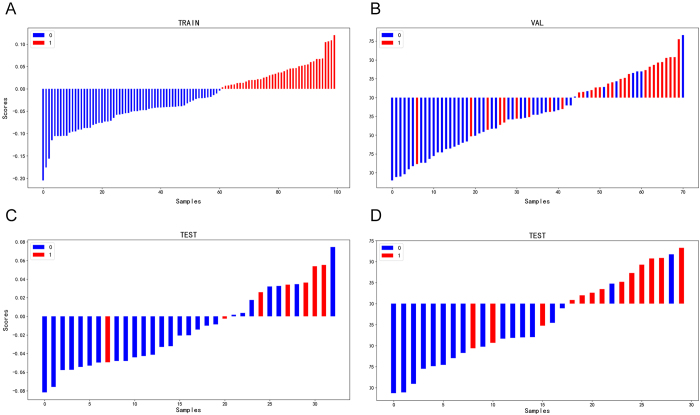
Rad-score waterfall plot of drug resistance in patients with LNTB.

The Rad-score of each patient was described in a box chart. There was a significant difference between the Rad-score of LNTB resistant and sensitive patients in the neck (*P* < 0.001), as shown in Figure 3. In addition, the model’s precision-recall curves were mapped to further evaluate the model’s performance in recovering drug-resistant cases, providing a more comprehensive assessment of its diagnostic effectiveness, as shown in Supplementary Digital Content, Material 4 (available at: http://links.lww.com/JS9/E494).

(a) Training set and (b) internal validation set, (c) External validation set (Test A), and (d) External validation set (Test B), respectively. Red marks represent patients with resistant neck LNTB, blue marks represent patients with sensitive neck LNTB patients

### Model effectiveness evaluation

**Figure 4.**
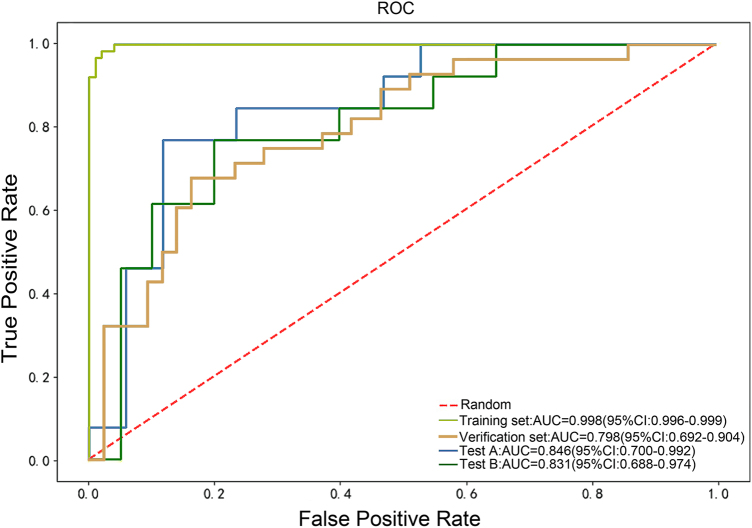
Receive operating characteristic curves of the prediction model.

**Figure 5.**
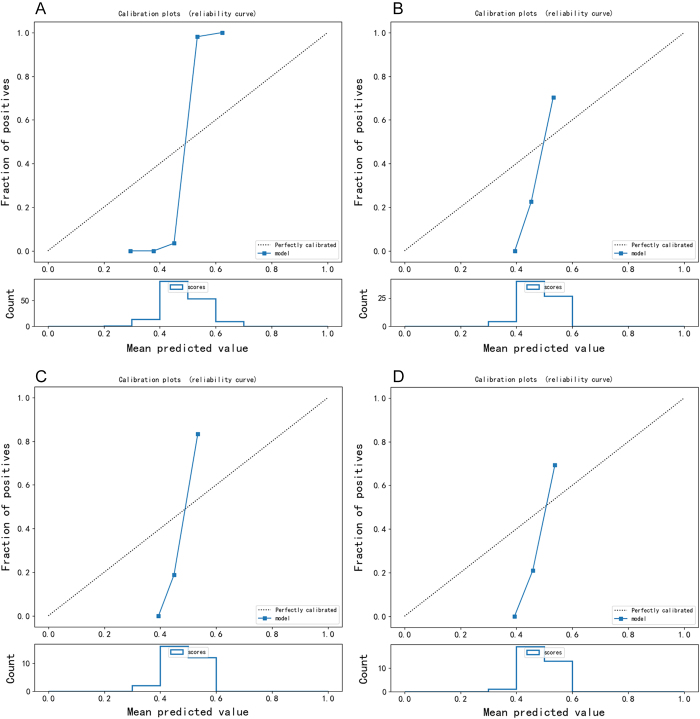
Calibration curves for the prediction model.

An internal validation queue and two external test queues were used to evaluate the diagnostic efficiency of the predicted model constructed by radiomics. An internal validation queue is a validation performed on a training dataset to evaluate the performance of a model on a relatively small sample set. The training set AUC and validation set AUC are obtained by plotting the ROC curves as shown in Figure 4 and Table 2. In addition, we generated calibration plots to assess the reliability of the predicted probabilities (Fig. 5). Calibration plots show the relationship between predicted probabilities and actual observed frequencies, providing insights into the model’s calibration performance.

Training set, internal validation set, external validation set (Test A), and external validation set (Test B), respectively.

(a) Training set and (b) internal validation set, (c) external validation set (Test A), and (d) external validation set (Test B), respectively. Calibration plots show the relationship between predicted probabilities and actual observed frequencies, providing insights into the model's calibration performance.

**Table 2:** Diagnostic performance of the predictive models

Group	AUC (95% CI)	ACC	Precision	Recall	F1_score
Training cohorts	0.998 (95% CI: 0.996–0.999)	0.975	0.983	0.953	0.968
Validation cohorts	0.798 (95% CI: 0.692–0.904)	0.760	0.703	0.678	0.690
Test A	0.846 (95% CI: 0.700–0.992)	0.833	0.833	0.769	0.800
Test B	0.807 (95% CI: 0.688–0.974)	0.818	0.769	0.769	0.769

Abbreviations: ACC, accuracy; AUC, area under the curve; CI, confidence interval.

Training set: AUC = 0.998 (95% CI: 0.996–0.999), ACC = 0.975, precision = 0.983, recall = 0.953, F1 score = 0.968.

Verification set: AUC = 0.798 (95% CI: 0.692–0.904), ACC = 0.760, precision = 0.703, recall = 0.678, F1 score = 0.690.

To further test the diagnostic effectiveness of the model, an external test cohort is used to assess the generalization ability of the model. The results of the external Test set (Test A, Test B) are as follows.

Test A: AUC = 0.846 (95% CI: 0.700–0.992), ACC = 0.833, precision = 0.833, recall = 0.769, F1 score = 0.800.

Test B: AUC = 0.831 (95% CI: 0.688–0.974), ACC = 0.818, precision = 0.769, recall = 0.769, F1 score = 0.769.

The AUC for Test A (0.846) was marginally higher than that for Test B (0.831). This difference can be attributed to variations in sample characteristics and distribution between the two cohorts. To further assess the generalizability of our model, we conducted a statistical comparison between the internal validation set and the external test sets (Test A and Test B). The results showed no significant differences in AUC values between the internal validation set and the external test sets (*P* > 0.05), indicating that the model maintains consistent performance across different datasets.

**Figure 6.**
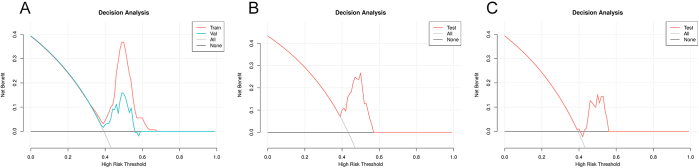
Decision curve analysis (DCA) for the prediction model.

In the decision curve, the results show that the model is within the threshold probability range of 0.38 to 0.57 in the validation set, and the predicted model has a large net benefit, as shown in Figure 6. By comparing directly with existing diagnostic methods, such as Xpert/RIF, our model could avoid 17 unnecessary biopsies in Test A and Test B, thereby reducing patient morbidity and health care costs. This indicates that when the predicted probability of drug resistance falls within this range, the model provides the greatest clinical utility. Specifically, clinicians can use this information to guide treatment decisions.

(a) Training set and internal validation set, (b) external validation set (Test A), and (c) external validation set (Test B), respectively.

## Discussion

In this multi-center study, we developed and validated an ultrasound radiomics-based predictive model for LNTB drug resistance. In this study, an ensemble machine learning scoring prediction model is used for the first time to predict LNTB resistance. This model exhibits high diagnostic efficiency in both the independent internal validation queue and the external test queue. The implementation of non-invasive diagnosis of LNTB resistance provides a new perspective and approach to the study of resistance mechanisms.

As a non-invasive and quantitative diagnostic method, radiomics has many advantages over traditional drug susceptibility test or molecular detection (such as Gene Xpert MTB/RIF) of specimens after invasive tissue biopsy^[21,22]^. Image group learning can convert video image to high-dimensional data, in-depth in noninvasive way to capture a lot of naked eye cannot identify the characteristics of disease. The biological relevance of wavelet-transformed texture features (e.g., necrotic heterogeneity) to drug resistance mechanisms (e.g., mycobacterial cell wall alterations) is hypothesized based on prior studies^[23,24]^.

For instance, drug-resistant strains may induce chronic inflammation, leading to tissue fibrosis and heterogeneous necrosis, which could be reflected in ultrasound texture through wavelet-based feature extraction. While direct histopathological validation was beyond the scope of this study, these findings provide a rationale for future mechanistic investigations^[25,26]^. A CT study of multidrug-resistant tuberculosis found that multidrug-resistant tuberculosis had the characteristics of severe lesions, obvious activity and poor treatment effect, indicating that imaging characteristics of tuberculosis may be related to drug resistance after anti-tuberculosis treatment^[27]^. Jaeger^[28]^ *et al* extracted the chest X-ray features of drug-resistant and sensitive pulmonary tuberculosis patients, and used MATLAB standard pattern recognition neural network and SVM and other algorithms to find that the model based on radiomics features had the potential to diagnose drug resistance in patients. The above studies suggest that there are some differences in chest imaging characteristics between sensitive and drug-resistant tuberculosis, and it is possible to evaluate drug resistance after anti-tuberculosis treatment by imaging characteristics.

However, there are few studies on the application of ensemble machine learning in the imaging diagnosis of drug-resistant extrapulmonary tuberculosis, and there are few reports on the radiomics study of LNTB drug resistance at home and abroad^[13,29]^. In this study, ultrasound images of drug-resistant or sensitive LNTB in the neck were collected, and AdaBoost classifier based on ensemble machine learning was used to construct a drug resistance prediction model to identify the drug resistance of LNTB. The AdaBoost algorithm is a lifting method by combining multiple weak classifiers to form a strong classifier^[30]^. In the training process, the algorithm paid more attention to weighting the appropriate sample, the sample of error. By repeating this process, the samples were correctly classified. Then, the final prediction result is determined by a majority vote to improve the accuracy of classification^[31,32]^. The drug resistance diagnostic model constructed in this study performed well in the internal validation set and the external test set, indicating that the radiomics model has a certain value in diagnosing the drug resistance of LNTB.

Generalization ability is one of the important metrics to evaluate model performance. It measures the model’s ability to adapt to the new sample and prediction ability, not just on the training data well^[33,34]^. In this study, the accuracy, sensitivity and specificity of the prediction model in the training set were all over 90%, but overfitting occurred in the external test set, and its generalization ability was weak. Overfitting is usually caused by too complex a model, too small a training set or too many training times. When the model is too sensitive to noise and specific sample features in the training set, and even the noise in the training set is learned, the model will be overfitted, and its performance on the verification set may be significantly reduced^[35,36]^. In addition, if the size of the training set is small, the model’s memory capacity to the training samples is also prone to overfitting, so it cannot be well generalized to new samples^[37]^.

In order to further solve the model overfitting problem, the penalty parameters introduced in the loss function of the model can be considered to be optimized in future research to limit the complexity of the model and make it prefer simple solutions, thus reducing the risk of overfitting^[38]^. In addition, the complexity of the model can be reduced by selecting the most relevant or useful features, reducing the risk of overfitting. At the same time, increasing the size of the training data set can improve the generalization ability of the model, thereby reducing the influence of overfitting^[39]^.

To further address the issue of overfitting and improve the generalization of our model, several strategies could be considered in future research^[40]^. First, data augmentation techniques could be employed to increase the diversity of the training dataset by generating synthetic samples through operations such as image rotation, scaling, and cropping. Second, cross-validation methods, such as k-fold cross-validation, could be implemented to more comprehensively evaluate the model’s performance across different subsets of data. Lastly, regularization techniques, including L2 regularization and Dropout, could be incorporated into the model to limit its complexity and reduce the risk of overfitting^[41]^.

The slight difference in AUC between Test A and Test B suggests that the model’s performance may be influenced by the specific characteristics of the patient populations in each cohort. Future studies should aim to further explore these differences and identify any potential biases or confounding factors that may affect model generalizability. The differences in performance between different external test groups, such as the lower F1 score in Test B [0.769 vs. 0.800 in Test A], may be related to variations between ultrasound systems, such as the difference in spatial resolution between Philips EPIQ and GE Logiq. Although we performed image normalization at an early stage, transducer frequency may reduce spatial resolution, potentially blurring texture details critical for radiomic feature extraction. Additionally, gain settings varied across centers, which may have caused uneven grayscale distributions. These factors highlight the need to establish standardized acquisition protocols to improve feature reproducibility across different centers.

In order to further evaluate the clinical value of the diagnostic model, using clinical DCAs to quantify the net benefit of different threshold probabilities, the forecasting model within the scope of the threshold probability 0.38 0.57 has the greatest net benefit. The DCA results provide valuable insights into the clinical applicability of our predictive model. The significant net benefit observed within a specific threshold probability range suggests that the model can effectively support clinical decision-making^[42]^. When the predicted probability of drug resistance is within this range, clinicians can leverage the model’s predictions to optimize treatment plans, ensuring patients receive the most appropriate care based on their individual risk profiles. Radiomics has a certain potential in the diagnosis of LNTB drug resistance. It is limited by the number of drug-resistant LNTB cases, and only on the basis of conventional ultrasound image characteristics, the subsequent need to further develop related data collection and tagging work, to enhance the effectiveness of the model.

The clinical utility of our radiomics-based prediction model should be further developed. Compared to existing diagnostic methods such as invasive tissue biopsies and molecular assays (e.g., Gene Xpert MTB/RIF), our model has several advantages in cost, accessibility, and integration into clinical workflows^[43]^. It relies on conventional ultrasound imaging, which is widely available and does not require additional resources. This makes it cost-effective and suitable for resource-limited settings. In addition, it can generate predictions immediately after an ultrasound examination, providing timely diagnostic information to complement traditional methods and enhance clinical decision-making.

The limitations of this study are: (1) sample size is small, need to be in a follow-up study to accumulate more cases to optimize the model, the effectiveness of clinical diagnosis of this learning model of image will in the future with more characteristics of tested in clinical trials; ⑵ This study only focused on gray-scale ultrasound, while the radiomics model is better to extract more quantitative data from multimodal ultrasound images, such as blood flow ultrasound, elastic ultrasound and CEUS; ⑶ The composite diagnostic model was not constructed by combining clinical indicators. In future research, we intend to collect multimodal imaging data and develop a joint diagnostic model to improve the accuracy of predicting drug resistance in LNTB. Proposed extensions involve fusing elastography strain ratios or serum interferon-γ (IFN-γ) levels with B-mode radiomic features to increase the AUC beyond 0.90. Collaborative refinement with radiologists, statisticians, and tuberculosis specialists will ensure the model yields clinically actionable interpretations. Although the methodology presented here is novel and shows enhanced diagnostic performance compared to traditional methods, the limited sample size necessitates further investigation. Prospective studies are essential to externally validate the model and incorporate a larger cohort of drug-resistant LNTB cases, thereby strengthening its predictive utility.

## Conclusion

The LNTB resistance prediction model developed in this study showed high diagnostic efficacy in an independent internal validation cohort and an external test cohort. Radiomics can provide more potential information and new perspectives for the study of drug resistance mechanisms. It can provide patients with more effective treatment and provide potential methods and strategies for the solution to drug resistance.

## Data Availability

To protect patient privacy, pathology image datasets and other patient-related data were not publicly accessible. However, all data were available upon reasonable request from the corresponding author. To gain access, data requests must sign a data access agreement.

## Research registration unique identifying number (UIN)

This study has been registered with the Chinese Clinical Trial Registry under the registration number ChiCTR2100051732.
